# Multidisciplinary Postoperative Ileus Management: A Narrative Review

**DOI:** 10.3390/medicina61081344

**Published:** 2025-07-25

**Authors:** Sun Yu, Katrina Kerolus, Zhaosheng Jin, Sandi Bajrami, Paula Denoya, Sergio D. Bergese

**Affiliations:** 1 Department of Surgery, Stony Brook University Renaissance School of Medicine, Stony Brook, NY 11794, USA; sunjyu325@gmail.com (S.Y.); paula.denoya@stonybrookmedicine.edu (P.D.); 2 Department of Anesthesiology, Stony Brook University Renaissance School of Medicine, Stony Brook, NY 11794, USA; katrina.kerolus@stonybrookmedicine.edu (K.K.); sergio.bergese@stonybrookmedicine.edu (S.D.B.); 3 Renaissance School of Medicine, Stony Brook University, Stony Brook, NY 11794, USA; sandi.bajrami@stonybrookmedicine.edu

**Keywords:** enhanced recovery pathways, gastric motility, neurogenic inflammation, opioid-sparing analgesia, perioperative management, postoperative ileus, surgical outcomes

## Abstract

Postoperative ileus is a prolonged impairment of gastrointestinal motility following surgical procedures. This often leads to increased morbidity, extended hospital stays, and high healthcare expenditures. In this review, we discuss the pathophysiology underlying postoperative ileus, its epidemiology, and perioperative management strategies. Patient characteristics, as well as expected perioperative course, could be used to stratify the risks of postoperative ileus. Preventive measures hinge upon a multimodal approach, minimally invasive surgical techniques, fluid management, early postoperative ambulation, and opioid-sparing analgesia strategies. Adjuvant interventions such as alvimopan, caffeine, and chewing gum have demonstrated efficacy in modulating the neurogenic and inflammatory components of postoperative ileus. Minimally invasive approaches, comprehensive perioperative management, and adjuvant therapies hold promise for prevention. Current management relies heavily on supportive care, underscoring the need for research into the underlying neurogenic and inflammatory mechanisms to guide the development of targeted treatments.

## 1. Introduction

Postoperative ileus (POI) refers to the slow return of bowel transit postoperatively. Although it remains poorly defined, it is well understood that POI increases length of stay, morbidity, and, therefore, healthcare costs. The pathophysiology of postoperative ileus is thought to be multifaceted, mainly neurogenic and immunogenic in nature. Many associated perioperative factors have been identified, thus requiring a multidisciplinary approach to optimally manage. In this narrative review, we explore the pathophysiology, prevention, and treatment of postoperative ileus.

We conducted a comprehensive literature search on PubMed and Medline using search terms including “postoperative ileus”, “prevention”, “surgical technique”, and “anesthesia”. Publications were selected based on the novelty, quality, and relevance to the discussion. The findings of the literature are summarized as a narrative review, which covers current basic science evidence on the pathophysiology of postoperative ileus; as well as clinical evidence on the perioperative management of postoperative ileus.

## 2. Epidemiology

Some of the challenges in studying postoperative ileus arise as there is no clear clinical definition or timeline provided for POI. In abdominal surgeries, a certain degree of dysmotility is accepted as physiologic, and it is often difficult to define at which point the “physiologic” ileus becomes “pathologic”. One study reports that the most used cutoff is at least 4 days following the index surgery [1]. However, according to a meta-analysis of POI in patients who underwent colorectal surgeries, the cutoff threshold varies from 3 to 7 days, and some even defined POI simply as the need for nasogastric tube reinsertion for those who were perioperatively decompressed with a nasogastric tube [2]. Some advocate categorizing them as primary versus secondary POI to differentiate POI due to underlying disease processes such as intraabdominal abscesses or peritonitis [3]. The current lack of a standardized definition for this costly complication limits the accurate assessment of its clinical and financial impact, which should be addressed.

The reported incidence of POI varies widely from 8 to 21% in colorectal surgeries and 10 to 30% in abdominal surgeries due to the varying definition [2,3]. Regardless of its exact definition, POI is a condition that imposes a significant increase in healthcare costs. A cost-analysis study in Australia showed a 26% increase in hospital costs among colorectal surgery patients with postoperative ileus, mainly attributable to increased length of hospital stay [4]. The rate of POI diagnosis in this population was about 35%. The same group of authors performed a meta-analysis of the effects of POI on global costs, with six studies involving over 170 thousand patients who underwent abdominal surgeries showing there was a 66.3% increase in total hospital costs [5]. The rate of POI diagnosis in these studies ranged widely from 3 to 35%, again likely due to varying definitions. Again, shown in this study was the predominant role of length of stay is in the increased cost. Interestingly, the authors of this study also noted a pattern of underestimation/underreporting of POI in studies that used ICD-9 code instead of a clinical definition, which led to such a wide range of POI diagnoses, again emphasizing the need for a standardized definition for POI [5].

Some of the risk factors suggested for POI for abdominal surgeries include male sex, advanced age, cardiac comorbidities, previous abdominal surgeries, an open approach to surgery, increased duration of surgery, significant blood loss or blood transfusion during surgery, and delayed mobilization after surgery [1,3,6]. According to a meta-analysis, male patients had a significantly higher rate of POI with an odds ratio (OR) of 1.43 compared to female patients [1]. It may be suggested that the reason for this difference may be due to the higher rate of visceral obesity. In a study involving patients with colorectal cancer undergoing operative interventions, visceral obesity was shown to be an independent preoperative risk factor for POI [7]. Authors of this study suggest that higher amounts of visceral adipose tissue put these patients at a higher risk of poorly regulated inflammatory response after bowel manipulation, as a study of human visceral adipose tissue has shown it to be a major contributor to increased levels of inflammatory cytokines [8]. This, combined with bowel manipulation, may lead to a higher level of local and systemic inflammatory response (which is one of the mechanisms described below) than those without visceral obesity, which may lead to an increased risk of POI. Patients of advanced age (>65 years) have a higher incidence of medical comorbidities that may also be risk factors for POI, such as cardiac comorbidities and previous abdominal surgeries. Some of modifiable factors, such as surgical approach, intraoperative blood loss, and delayed mobilization after surgery, are explored in the later section discussing preventive measures.

POI is associated with several significant adverse outcomes. As POI delays the return of bowel function and limits the initiation of oral intake, it delays discharge readiness, leading to increased healthcare costs associated with the extended inpatient care, possible additional diagnostic studies. Prolonged hospitalization secondary to POI may also increase the risk of hospital-acquired infections and venous thromboembolism, which further increases morbidity and healthcare resource utilization. In fact, studies have shown that POI can significantly increase healthcare costs even when it is transient [9,10]. These studies underline the importance of minimizing the incidence and duration of POI.

## 3. Pathophysiology

**Figure 1:**
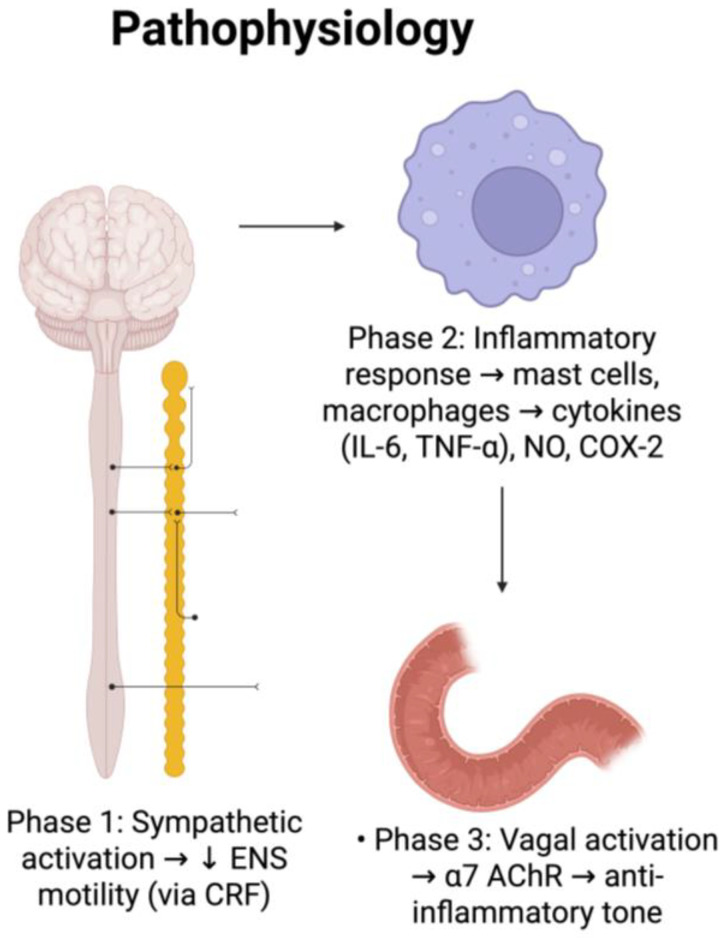
Summary of pathophysiology of postoperative ileus.

The pathophysiology of POI is believed to be multifactorial and involves various mechanisms including neurogenic, inflammatory, and pharmacological factors. It affects different segments of the gastrointestinal tract with small intestine motility first during the first 24 h, gastric motility from 24 to 48 h, and colonic motility from 48 to 72 h postoperatively [11]. According to the current literature, POI likely occurs in three phases: neurological phase via sympathetic activation, hormonal and inflammatory phase, and resolution phase via parasympathetic nervous activation (Figure 1)

Normal motility is regulated by the enteric nervous system, which is found within the lining of the gastrointestinal system. The enteric nervous system (ENS) comprises more than 200–600 million neurons with 20 different neuron types, two plexuses, muscle types, and cells to control motor functions and regulate local blood flow, mucosal transport, and secretion. The ENS is composed of two main types of neurons: intrinsic primary afferent neurons (IPANs) and enteric motor neurons. The neurons are collected into two types of ganglia: the myenteric and the submucosal plexuses. The myenteric plexus works to increase the tone of the gut and the velocity and intensity of contractions, while the submucosal plexus controls local conditions, secretion, and absorption. When there are changes in the gut, such as the presence of food, distension, chemical stimuli, or changes in pH, IPANs are stimulated to detect the changes. They forward this information throughout the ENS and to the central nervous system (CNS) via the vagus nerve [12].

The first phase of POI is the neurological phase, which involves the sympathetic nervous system. Different neural pathways can be activated based on the intensity of the stimulus and the location of the intestine. Skin incisions and laparotomy briefly cause immobility by the activation of the adrenergic inhibitory pathway via afferent splanchnic nerves synapsing in the spinal cord [13]. More aggressive stimuli, such as the handling of the intestines, trigger an additional supraspinal pathway found in the brainstem. This pathway is mediated by the release of corticotropin-releasing factor (CRF). It is proposed that the release of CRF stimulates neurons in the supraoptic nucleus of the hypothalamus, which then interact and activate sympathetic preganglionic neurons to cause an acute intestinal paralysis [10,14,15]. These processes, however, cease following the closure of skin and the end of the procedure.

The second phase of POI is the inflammatory phase. This typically occurs three to four hours following the first surgical incision and is regulated by proinflammatory cytokines and chemokines. This phase is prolonged and is responsible for much of the ileus that occurs; therefore, prevention of this phase would be most effective in treating postoperative ileus. Key players include peritoneal mast cells and macrophages. With manipulation, mast cell mediators such as substance P and calcitonin gene-related peptide are released from afferent nerve cells, which are in close proximity to mast cells in the mesentery. This will lead to the release of vasoactive substances like histamine that increase intestinal permeability with translocation of intraluminal bacteria and their products. Afterward, these products will be phagocytosed through macrophages. There are multiple ways of triggering an increase in macrophage response, which include the release of DAMPs (molecules that are released in response to cell damage), cytokines, and the translocation of bacteria. When macrophages in the muscularis externa are activated, they lead to production of chemokines, proinflammatory cytokines (TNF-alpha and IL-6), and integrins [16]. With the release of cytokines and chemokines, there is an upregulation of intracellular adhesion molecules (ICAM-1), which trigger the recruitment of neutrophils and monocytes to the site of injury [17]. Memory T cells are activated and disseminated via the lymphatic system, causing a systemic inflammation and POI [18].

Phosphorylation of transcription factors results in secretion of cytokines and chemokines, such as nitric oxide (NO), prostaglandins, reactive oxygen intermediates, and defensins. This occurs via alpha 2 adrenergic receptors in the inflamed mucosa that lead to increased synthesis of mRNA for nitric oxide synthetase that releases NO. Throughout the gastrointestinal tract, NO works predominantly as an inhibitory noradrenergic noncholinergic neurotransmitter. Also, NO can lead to activation of cyclooxygenase 2 (COX-2), which is responsible for the release of prostaglandins into the peritoneal cavity. It was seen that there was a decrease in jejunal circular muscle contractility in a study conducted on mice as a result [19].

The final phase is the resolution phase and is controlled by the vagal system. Activation of 5-hydroxytryptamine 4 receptors results in increased acetylcholine receptors. This then allows for the activation of nicotinic alpha 7 acetylcholine receptors on macrophages and works to reduce the inflammatory response. Because of this cascade, it has been proposed that chewing gum can be helpful because it helps to stimulate vagal tone and thus enhance anti-inflammatory properties [20].

In addition to physiological causes of ileus, there are also pharmacological causes. Most commonly, the use of opioids will lead to ileus via peripheral μ and κ receptors found in the gastrointestinal tract. Opioid receptors are G-coupled protein receptors present in smooth muscle cells and on the terminals of sympathetic and sensory peripheral neurons [21]. The submucosal plexus is densely populated with μ receptors, while the myenteric plexus is highly populated on the myenteric plexus. These receptors are activated in small and large intestine by endogenous opioids, such as enkephalins, endorphins, and dynorphins, as well as exogenous opioids like morphine, oxycodone, and methadone. Opioids function through neurotransmitters such as Ach, NO, and vasoactive intestinal peptide, which affect neuronal excitability by inhibiting both excitatory and inhibitory neural pathways found within the ENS, ultimately leading to bowel dysmotility. Opioids also work on the submucosa to inhibit water and electrolyte secretion in the gut and increase reabsorption of fluid in the intestine, leading to dry, hard stools [22].

Psychotropic medications, such as tricyclic antidepressants (TCAs), selective serotonin reuptake inhibitors (SSRIs), serotonin-norepinephrine reuptake inhibitors (SNRIs), antipsychotics, and anxiolytics may also adversely affect gastrointestinal motility [23]. For example, TCAs, and certain antipsychotics and anxiolytics, block muscarinic receptors present on intestinal epithelial cells, leading to POI [24]. SSRIs and SNRIs affect the serotonin level, which plays a crucial role in gut motility through the enteric nervous system [23]. Furthermore, major depressive disorder is associated with significantly slower gastric emptying [25,26].

## 4. Preventative Strategies

### 4.1. Surgical Planning

**Figure 2:**
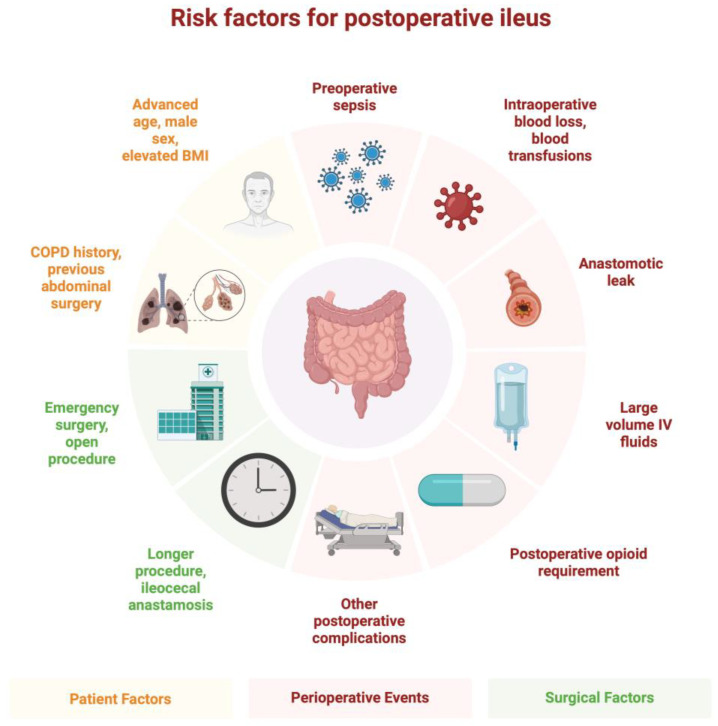
Summary of postoperative ileus risk factors.

Some of the modifiable risk factors for developing POI suggested by the current literature include delayed mobilization after surgery, significant blood loss during surgery, open approach to surgery, and increased duration of operation (Figure 2) [3]. The Enhanced Recovery After Surgery (ERAS) is a comprehensive perioperative care pathway developed to improve postoperative outcomes, with most of its recommendations based on data from patients undergoing colorectal surgeries. However, it has been applied across a broad range of surgical specialties including bariatric, hepatobiliary, gynecologic, orthopedic surgery, and more [27]. ERAS focuses on early return to normal function, and as current literature shows, early mobilization is beneficial to patient outcomes and reduces healthcare costs. Many elements of ERAS are focused on early activities [28]. For example, a recent prospective randomized controlled trial of 119 patients undergoing hepatic resection, early ambulation was significantly associated with early return of bowel function and decreased pain [29]. ERAS guidelines recommend routine, dedicated preoperative counseling and optimization [30]. Preoperative counseling is an opportunity for physicians to educate and encourage patients to take an active part in their recovery process. Although further study is warranted to understand the exact relationship between early mobilization and POI, as a multitude of studies indicate improvement of perioperative outcomes with early mobilization, the importance of early activities after surgery should be emphasized to patients during preoperative counseling session.

Current evidence suggests that excessive intraoperative bleeding may be a risk factor for POI [3]. A retrospective chart review of patients undergoing elective colorectal surgeries revealed that estimated blood loss was significantly associated with prolonged POI, even after adjusting for operative duration [31]. The authors of this study posit that the increased level of sympathetic and endocrinologic response to blood loss during surgery may cause increased levels of inflammatory cytokines, which may lead to prolonged POI. Therefore, during preoperative optimization, multidisciplinary discussions should take place regarding the cessation of any anticoagulant or antiplatelet agents for applicable patients, weighing the risks and benefits for each patient’s unique clinical status.

Given that inflammatory processes are believed to be contributory to POI, inflammatory markers such as IL-1, 6, TNF-alpha, and CRP have been suggested as predictors of POI [32]. Elevated preoperative neutrophil-to-lymphatic ratio (NLR), as well as uptrend in NLR after surgery, were associated with a higher risk of POI [33]. Low serum albumin or abnormalities in serum electrolytes are also associated with POI [34,35]. On the other hand, it is not clear how these biomarkers will perform at the population level for the purpose of POI risk stratification.

### 4.2. Surgical Approaches

**Figure 3:**
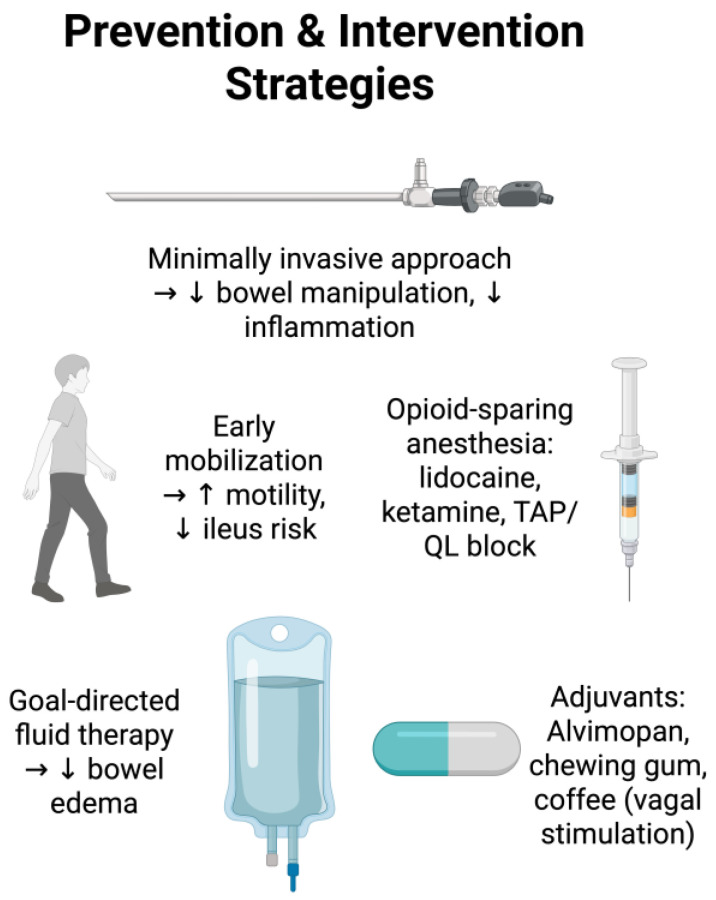
Summary of postoperative ileus prevention and treatment.

With the gaining popularity of minimally invasive surgery, endoscopic/laparoscopic/robotic procedures have become widely available, and these approaches may decrease the risk of postoperative ileus (Figure 3). Bowel manipulation induces an intramural inflammatory response, which has been implicated in the inflammatory phase of postoperative ileus [36]. Therefore, excessive manipulation of the bowel during operation can lead to prolonged gastrointestinal dysmotility, and approaches that minimize bowel manipulation may lead to a lower risk of postoperative ileus. Current literature supports this hypothesis. For example, studies have shown that laparoscopic colectomies are associated with lower rates of ileus compared to open colectomies [37,38]. Decreased rates of ileus were also reported in patients who underwent laparoscopic appendectomy or cholecystectomy when compared to open [38,39]. This result was also replicated in a study of abdominal aortic procedures comparing the transperitoneal versus the retroperitoneal approach, favoring the retroperitoneal approach [40]. Unnecessary and excessive bowel manipulation must be avoided, and in appropriate candidates, minimally invasive or retroperitoneal approaches should be considered, as improved visualization and maneuverability allow for decreased need for bowel handling, which may lead to earlier return of bowel function.

Routine use of nasogastric decompression or intra-abdominal drainage in patients undergoing abdominal surgeries has not been shown to improve the return of bowel function while increasing comorbidities, and, therefore, it is not recommended [41]. Randomized controlled studies of patients undergoing colorectal surgeries have shown that routine postoperative continuation of nasogastric drainage or intraoperative placement of peritoneal drains did not show any perioperative benefits, at the expense of drain-specific complications [41,42,43]. As discussed earlier, local or systemic inflammation triggered by intraluminal bowel injuries may contribute to the inflammatory phase of POI. Therefore, routine use of these measures with possible inflammatory consequences without proven benefits should be avoided.

### 4.3. Intraoperative Opioid-Sparing or Opioid-Free Anesthesia

It is generally accepted that balanced anesthesia with multimodal analgesia during the perioperative period is the best approach to minimizing side effects associated with opioids. This was an integral aspect of the original ‘fast-track’ surgery concept, which consisted of epidural analgesia and standardized perioperative analgesia [44]. Effective operative opioid-sparing strategies have demonstrated efficacy in reducing the risk of postoperative ileus. A meta-analysis of patients undergoing colorectal surgeries reported that intraoperative lidocaine significantly reduces the risk of postoperative ileus (OR 0.32) [45]. Similar findings have also been reported with ketamine [46] and magnesium [47].

The emergence of various non-opioid analgesic modalities has led to the conception of opioid-free anesthesia, this is usually achieved through a combination of regional anesthesia techniques, non-opioid analgesics, and pain adjuncts. While this practice is feasible, it is not clear if opioid-free anesthesia results in an overall better outcome than an opioid-sparing approach [48]. A multicenter clinical trial compared the use of opioid-free anesthesia with dexmedetomidine versus intraoperative remifentanil in combination with a multimodal perioperative analgesic regimen (intraoperative ketamine, intraoperative and postoperative lidocaine infusion, postoperative NSAIDs, and acetaminophen) [49]. This study was terminated prematurely due to an incidence of bradycardia with dexmedetomidine, interim analysis revealed no significant difference in the risk of POI between the two approaches. The use of lidocaine or dexmedetomidine infusion as a single intraoperative intervention had a marginal benefit on postoperative opioids, with no impact on the return of bowel function [50].

On the other hand, a clinical trial showed that when compared to intraoperative remifentanil infusion, a combination of lidocaine, ketamine, and dexmedetomidine infusion (opioid-free anesthesia) was associated with significantly lower opioid requirements as well as time to flatus [51]. Similarly, another small trial showed a lower risk of ileus in patients receiving magnesium, lidocaine, and ketamine infusion when compared to intraoperative fentanyl [52]. The above studies would support the use of multimodal analgesia to improve postoperative bowel function recovery; whereas complete avoidance of opioids may necessitate higher doses of other analgesics that have their own adverse effects.

### 4.4. Regional Anesthesia and Postoperative Ileus

The transverse abdominis plane (TAP) block targets the anterior rami of the spinal nerve and is commonly used to manage somatic pain from abdominal or pelvic surgeries (i.e., incision or port site pain). This could be performed as a single injection or with block catheters for continuous infusion. It could also be conducted intraoperatively by the surgeons under laparoscopic vision. In patients who underwent laparoscopic cancer resection, those who received a single-shot TAP block had significantly lower postoperative opioid requirements, and subsequently a shorter time to flatus [53,54]. Continuous TAP block has also been shown to effectively shorten the time to flatus after laparoscopic colorectal resection (31 vs. 41 h) [55]. Emile conducted an RCT, which compared no block, laparoscopic, and ultrasound-guided TAP blocks and reported that TAP block with either approach had a significantly shorter time to ambulation and time to flatus [56]. On the other hand, a meta-analysis of TAP block in patients undergoing colorectal resection (laparoscopic and open) reported that TAP block did not reduce the risk of overt postoperative ileus [57]. Additionally, TAP block may not reduce pain nor improve bowel function in patients undergoing open surgery [58].

In patients who underwent laparoscopic adrenalectomy, patients who received a single-shot quadratus lumborum (QL) block had significantly lower postoperative opioid requirement as well as shorter time to flatus (18.5 vs. 23.5 h) [59]. Similar findings have also been reported in patients undergoing gastrectomy [60,61]. In open liver resection, patients who received continuous QL catheter had significantly faster recovery of bowel function [62].

Hamid et al. [63] conducted a meta-analysis comparing TAP block with epidural analgesia after colorectal resection, including four laparoscopic colectomy studies and two mixed studies that included laparoscopic and open procedures. The authors reported no significant difference in the time to first flatus between epidural analgesia and the TAP block. Several other clinical trials that compare the effect of truncal blocks on the return of bowel function also reported comparable outcomes among different blocks [64,65].

Regional anesthesia techniques could shorten the time to return of bowel function, either by reducing postoperative opioid requirement or through neurohumoral modulation. However, it is not clear if regional anesthesia alone could reduce the incidence of clinically significant ileus.

### 4.5. Intraoperative Fluid Management

Judicious fluid management is a central aspect of perioperative care within abdominal surgery. It is thought that excessive fluid administration and subsequent bowel edema are major contributing factors in the development of postoperative ileus [66]. Several retrospective studies of patients undergoing rectal and bladder resections reported that patients who received more intraoperative fluids had a significantly higher risk of POI [66,67,68,69]. Interestingly, a small clinical trial of patients undergoing bowel resection and primary anastomosis found that patients who received colloid fluids had significantly shorter time to first flatus when compared to those who received crystalloids (73 vs. 84 h) [70]. This is reaffirmed in another small clinical trial, which reported that use of colloids resulted in significantly shorter time to flatus (2.4 vs. 3.5 days) as well as shorter time to first bowel movement (3.3 vs. 1.4 days) [71].

An earlier meta-analysis of patients undergoing surgery (excluding trauma and obstetric procedures) found that intraoperative goal-directed hemodynamic and fluid management (monitored using transesophageal doppler or arterial waveform analysis), resulted in shorter time to flatus (mean difference 0.37 days, favoring goal-directed fluid therapy) [72]. Another meta-analysis reported similar findings (mean difference 0.41 days) [73]. However, a more recent meta-analysis of patients undergoing non-cardiac surgery reported that use of goal-directed fluid therapy did not reduce the incidence of postoperative ileus (odds ratio 0.63–1.1, low quality evidence) [74].

## 5. Postoperative Considerations

### 5.1. Postoperative Analgesia

A large retrospective study found that in patients undergoing abdominal surgeries, patients who received more opioids in the postoperative period had a significantly higher risk of developing postoperative ileus [75]. In addition to regional anesthesia, effective opioid-sparing strategies include the use of acetaminophen [76], ketorolac [77], and ibuprofen [78].

### 5.2. Alvimopan and Postoperative Ileus

Alvimopan is a peripherally acting μ-opioid receptor antagonist with limited central nervous system penetration. This is thought to promote bowel motility without inducing systemic side effects. A recent clinical trial of patients undergoing cytoreductive surgery and hyperthermic intraperitoneal chemotherapy randomized patients to receive alvimopan 12 mg twice per day for 7 days versus a corresponding placebo; patients who received alvimopan had significantly shorter times to solid food intake (117 vs. 152 h) as well as shorter times to bowel movement (67 vs. 89 h) [79]. Additionally, a meta-analysis of patients undergoing cystectomy reported that alvimopan (12 mg twice per day up to a maximum of 15 doses or until initiation of oral intake) is associated with a significantly shorter time to oral intake as well as time to bowel movement [80]. The most common adverse effect of alvimopan is dyspepsia [81].

### 5.3. Acupuncture and Transcutaneous Electrical Nerve Stimulation

While the exact mechanism is not clear, acupuncture has consistently been shown to reduce nausea and vomiting after surgery. It has been suggested that acupoint stimulation could also promote bowel motility after surgery. In patients undergoing cystectomy, auricular stimulation significantly reduced the incidence of postoperative ileus (6% vs. 20%) [82]. Similar findings have also been reported in patients undergoing colorectal surgery who received electro-acupuncture at lower limb acupoints [83,84,85]. A meta-analysis reported that acupressure and electro-acupuncture significantly reduced the time to flatus, as well as time to bowel movement [45].

### 5.4. Chewing Gum and Postoperative Ileus

Although data are limited to colorectal surgeries and cesarian sections, postoperative chewing gum has been shown to shorten time to recovery from POI [86,87]. It has been suggested that this is secondary to cephalic vagal activation, leading to reduction of postoperative inflammation [88]. However, most studies are small and low in quality, and further investigation is warranted to elucidate the benefits and safety of routine use of chewing gum.

### 5.5. Coffee and Postoperative Ileus

An open-label randomized controlled study conducted in Switzerland on patients undergoing elective colorectal surgeries reported that caffeine resulted in earlier resumption of bowel function after surgery, as defined by the first postoperative bowel movement [89]. Other meta-analysis studies showed similar results with postoperative coffee intake improving rate of POI and hospital length-of-stay in patients undergoing colorectal or gynecologic procedures [90,91]. It is believed that caffeine reduces inflammatory effects in the gastrointestinal system by competitively antagonizing an adenosine receptor found on immune cells [92]. However, some studies showed that coffee consumption decreased time to bowel function postoperatively, regardless of presence of caffeine (analysis of use of decaffeinated coffee also yielded similar effects) [90]. Therefore, the clear roles of caffeine and coffee in their effects on bowel function remain unknown.

## 6. Treatment of Postoperative Ileus

As POI is a difficult condition to treat, with very little data from robust studies to guide therapy, prevention of POI with diligent preoperative assessment and planning with use of adjunctive means mentioned above is imperative to early return of bowel function. Supportive care, including adequate resuscitation, bowel rest, and decompression as necessary, is the mainstay of treatment for POI. Clinicians must consider the volume of emesis or nasogastric drainage, if any, to resuscitate adequately. One must also investigate and identify any underlying, reversible causes of POI and address them.

### 6.1. Prokinetic Agents

Prokinetic agents are often used to treat perioperative nausea and vomiting. Some of the examples of such agents are alvimopan (discussed earlier), metoclopramide, and erythromycin. Metoclopramide is a dopamine-2 and 5-HT3 antagonist, FDA-approved for the treatment of nausea and vomiting associated with gastroesophageal reflux, diabetic gastroparesis, and chemotherapy by decreasing the sensitivity of gastrointestinal afferent nerves that transmit signals to the chemoreceptor trigger zone [93]. Erythromycin, a macrolide antibiotic on the other hand, works on the motilin receptors, increasing gastric emptying by means of increased amplitude of antral peristalsis [94]. Although these agents are often used for the treatment of symptoms of postoperative ileus, studies regarding their effectiveness to shorten time to bowel function are small, low in quality, and inconclusive [95]. Therefore, they may be of value for temporary symptomatic management of postoperative ileus, but current literature does not support the use of these agents for improving outcomes associated with POI.

### 6.2. Non-Steroidal Anti-Inflammatory Drugs

As discussed above, the inflammatory phase of ileus is triggered by the activation of COX-2. Therefore, non-steroidal anti-inflammatory drugs (NSAIDs) have been studied as therapeutic agents for postoperative ileus. However, studies have failed to show consistent benefit in decreasing duration of POI with NSAIDs [96].

### 6.3. Propranolol and Postoperative Ileus

Antagonism of adrenergic receptors has been suggested as a treatment of POI, as the first phase of POI involves sympathetic activation. Propranolol (a nonselective beta-adrenergic receptor antagonist) has been studied in patients with postoperative ileus after abdominal surgeries, and results have been inconclusive, with a small study showing modest improvement in the duration of POI, while others showed no clinical improvement [97]. Further investigation is warranted.

## 7. Conclusions

POI poses significant healthcare costs with increased length of hospital stay postoperatively. The pathophysiology of POI is not yet clearly understood but is believed to involve mostly neurogenic and inflammatory responses to the stress of surgery. Given the scarcity of data regarding pharmacological targets, there are limited treatment options beyond supportive care for those who develop POI. Deliberate and thoughtful surgical planning with a multidisciplinary approach should be taken to prevent POI, especially in patients who are at high risk.

Using minimally invasive approaches when possible, avoiding unnecessary instrumentation of the peritoneum or bowel, minimizing intraoperative opioid use, judicious intraoperative fluid management, and use of postoperative nausea and vomiting prophylaxis may be of benefit to prevent POI. There are some nonpharmacologic practices suggested to prevent or treat POI, including chewing gum and coffee consumption, mostly relating to their ability to affect the neurogenic cause of POI. Studies involving pharmacologic agents, including prokinetics, NSAIDs, and beta-blockers, have yielded inconclusive data. POI is treated with supportive management with adequate fluid resuscitation, bowel rest, and bowel decompression as needed. If there are reversible causes underlying, they must be corrected to resolve secondary POI.

Given the healthcare outcome and economic impact of POI, it is imperative to develop and implement relevant pathways to best care for patients who are at significant risk. As with most perioperative multidisciplinary processes, effective implementation of pathways geared towards ensuring early return of bowel function, such as ERAS protocol, requires motivated personnel and a robust clinical informatic infrastructure. Lastly, more research is needed to elucidate the underlying biological process. A better understanding of its pathophysiology may facilitate the development of targeted treatments.

## Data Availability

Not applicable.

## Abbreviations

The following abbreviations are used in this manuscript:

CNS	Central nervous system
COX	Cyclooxygenase
CRF	Corticotropin-releasing factor
DAMPs	Damage-associated molecular patterns
ENS	Enteric nervous system
ERAS	Enhanced recovery after surgery
IL	Interleukin
IPANs	Intrinsic primary afferent neurons
NO	Nitric oxide
NSAIDs	Non-steroidal anti-inflammatory drugs
OR	Odds ratio
POI	Postoperative ileus
QL	Quadratus lumborum (block)
TAP	Transverse abdominis plane (block)
TNF	Tumor necrosis factor
